# Cutaneous manifestations of dermatomyositis characterized by myositis-specific autoantibodies

**DOI:** 10.12688/f1000research.20646.1

**Published:** 2019-11-21

**Authors:** Naoko Okiyama, Manabu Fujimoto

**Affiliations:** 1 Department of Dermatology, Faculty of Medicine, University of Tsukuba, Tsukuba, Ibaraki, 305-8575, Japan; 2 Department of Dermatology, Integrated Medicine, Graduate School of Medicine, Osaka University, Suita, Osaka, 565-0871, Japan

**Keywords:** dermatomyositis, myositis-specific autoantibodies

## Abstract

Dermatomyositis (DM) is an inflammatory myopathy with characteristic skin manifestations, the pathologies of which are considered autoimmune diseases. DM is a heterogeneous disorder with various phenotypes, including myositis, dermatitis, and interstitial lung disease (ILD). Recently identified myositis-specific autoantibodies have been associated with distinct clinical features. For example, anti-melanoma differentiation-associated protein 5 antibodies have a high specificity for clinically amyopathic DM presenting rapidly progressive ILD. Furthermore, anti-transcriptional intermediary factor 1γ antibodies found in patients with juvenile and adult DM are closely correlated with malignancies, especially in elderly patients. Finally, patients with anti-aminoacyl-transfer RNA synthetase antibodies share characteristic clinical symptoms, including myositis, ILD, arthritis/arthralgia, Raynaud’s phenomenon, and fever; thus, the term “anti-synthetase syndrome” is also used. With a focus on the characteristic cutaneous manifestations in each subgroup classified according to myositis-specific autoantibodies, we introduce the findings of previous reports, including our recent analysis indicating that skin eruptions can be histopathologically classified into myositis-specific autoantibody-associated subgroups and used to determine the systemic pathologies of the different types of antibody-associated DM.

## Introduction

Dermatomyositis (DM) is an inflammatory myopathy with characteristic skin manifestations, the pathologies of which are considered autoimmune diseases. DM is a heterogeneous disorder with various phenotypes, including myositis, dermatitis, and interstitial lung disease (ILD) ^1^. Recently, in addition to the already-established anti-aminoacyl-transfer RNA synthetase (ARS) antibody, a number of myositis-specific autoantibodies—including anti-melanoma differentiation-associated protein 5 (MDA5) antibody and anti-transcriptional intermediary factor 1γ (TIF1γ) antibody—that are not detected in patients with an inherited muscle disease ^2^ have been identified. These autoantibodies not only are highly disease-specific but also are associated with distinct clinical features ( Table 1) ^3, 4^. This article reviews their epidemiology and characteristic clinical features, with a focus on their characteristic cutaneous manifestations, to determine the systemic pathologies of the different types of antibody-associated DM.

**Table 1.** Clinical features and cutaneous manifestations characterized by myositis-specific autoantibodies.

Autoantigen	Clinical features	Typical cutaneous manifestations
MDA5	Clinically amyopathic DM * with ILD ^†^, especially rapid progressive ILD	Palmar violaceous macules/papules due to vascular injury
TIF1	Juvenile DM *; cancer-associated DM *	Severe cutaneous manifestations
Mi2	Classic DM *	Sometimes refractory
ARS	Anti-synthetase syndrome with chronic ILD ^†^	Mechanic’s hands
NXP2	Juvenile DM and adult DM	Calcinosis
SAE	Clinically amyopathic DM * followed by severe myositis including dysphagia	Extensive rash, sometimes as erythroderma

ARS, aminoacyl-transfer RNA synthetase; MDA5, melanoma differentiation-associated protein 5; NXP2, nuclear matrix protein 2; SAE, small ubiquitin-like modifier activating enzyme; TIF1, transcriptional intermediary factor 1. *Dermatomyositis; ^†^interstitial lung disease.

## Epidemiology and characteristic clinical features of subgroups classified according to myositis-specific autoantibodies

Anti-MDA5 antibody has a high specificity for clinically amyopathic DM (CADM) presenting rapidly progressive ILD (RP-ILD) ^5^. Anti-MDA5 antibody was first reported as an anti-CADM-140 antibody that reacted with a 140-kDa cytoplasmic protein ^6^ subsequently identified as the retinoic acid-inducible gene I (RIG-I)-like receptor MDA5/IFIH1 (interferon [IFN] induced with helicase C domain protein 1). The anti-MDA5 antibody is detected at high frequencies among patients with DM in Asia (15.8% [26/165 cases] in Japan and 36.6% [53/145 cases] in China) ^7^ and South America (16% [21/131 cases] in Brazil) ^8^ and at low frequency (2.8%, 21/748 cases) in a cohort of patients with DM in a combined European cohort in which 87.4% of enrolled cases were Caucasian ^9^. Case series studies reported CADM frequencies of around 40% in anti-MDA5 antibody-positive patients with DM ^7, 10^. A meta-analysis of 16 studies estimated pooled sensitivity and specificity of anti-MDA5 antibody for RP-ILD of 77% (95% confidence interval [CI] 64–87%) and 86% (95% CI 79–90%), respectively, with a pooled diagnostic odds ratio of 20.41 (95% CI 9.02–46.20) ^11^. The severity and prognosis of RP-ILD in anti-MDA5 antibody-positive patients with DM were strongly correlated with anti-MDA5 antibody titer (detected by established enzyme-linked immunosorbent assay) and serum ferritin level ^12^. In a series of 44 Japanese patients with juvenile DM (JDM), 41% were positive for anti-MDA5 antibody ^13^ compared with 7.4% of 285 patients with JDM in the UK ^14^. Both studies reported anti-MDA5 antibody to be strongly associated with ILD; however, only 8 (18%) of the 44 Japanese cases and none of the UK patients developed RP-ILD. A recent cohort study in the UK observed low myositis severity scores depending on muscle biopsies in 11 anti-MDA5 antibody-positive patients ^15^.

The anti-TIF1 antibody was originally described as anti-155/140 and anti-p155 antibodies targeting a 155-kDa nuclear protein, sometimes with a 140-kDa protein ^16, 17^. These antigens were subsequently identified as TIF1 family proteins belonging to the tripartite motif (TRIM) superfamily, TIF1γ (TRIM33) and TIF1α (TRIM24), respectively. Anti-TIF1γ antibody was detected in both adult DM and JDM patients and was closely correlated with malignancies, especially in elderly patients ^18– 20^, at high risk of dysphagia ^21^ and at low risk of ILD, Raynaud phenomenon, and arthritis/arthralgia ^22^. Anti-TIF1γ antibody was present in 7 to 15% of patients with DM ^9, 23^. A meta-analysis including 1,962 patients with DM demonstrated a prevalence of malignancy-associated DM of 0.41 in patients with anti-TIF1γ autoantibody (95% CI 0.36–0.45). The diagnostic odds ratio of cancer was 9.37 (95% CI 5.37–16.34) with low heterogeneity—Cochran’s Q, 14.88 (degrees of freedom = 17, *P* = 0.604), I ^2^ = 0%—in the presence of anti-TIF1γ autoantibody ^24^. In contrast, 30% of patients with JDM present anti-TIF1γ antibody ^17, 25^ and do not develop malignancies.

Patients with anti-ARS antibodies, including anti-Jo-1, anti-PL-7, anti-PL-12, anti-EJ, anti-OJ, anti-KS, anti-Ha, and anti-Zo, share characteristic clinical symptoms such as myositis, ILD, arthritis/arthralgia, Raynaud’s phenomenon, and fever; thus, the term “anti-synthetase syndrome” is also used ^26^.

The anti-Mi-2 antibody is directed mainly to Mi-2β, a component of the nucleosome-remodeling deacetylase complex ^27^. Anti-Mi-2 antibody was detected in 3% of patients with JDM ^25^ and 12% of patients with adult DM ^9^. Anti-Mi-2 antibody-positive patients have a lower risk of ILD and typically respond well to therapy, although the recurrence of DM symptoms is possible ^23^.

The anti-nuclear matrix protein 2 (NXP2) antibody, originally termed anti-MJ antibody, was first identified in a cohort of patients with JDM/juvenile polymyositis (JPM). Generally, anti-NXP2 antibody-positive myopathy is related to either DM or polymyositis (PM) phenotypes. Cohort studies have detected anti-NXP2 antibody in 22 to 25% of patients with JDM ^25, 28^. Another cohort study reported that severe myopathy characterized by muscle contractures and atrophy was associated with anti-NXP2 antibody-positive JDM ^28^. In contrast, anti-NXP2 antibody was detected in only 2.3% of patients with adult PM/DM ^9^. Moreover, two cohort studies of patients with adult PM/DM in Japan and the US suggested a possible association between anti-NXP2 antibody and malignancy ^19, 29^.

The anti-small ubiquitin-like modifier activating enzyme (anti-SAE) antibody, which was observed in about 6% of patients with DM ^9^, is associated with inflammatory myopathy with extensive rash and dysphagia ^30, 31^. The target autoantigen is a heterodimer of SAE1 (40 kDa) and SAE2 (90 kDa). ILD and malignancies were observed in, respectively, 42 and 21% of 46 previously reported patients with anti-SAE antibody-associated DM ^31^.

## Characteristic cutaneous manifestations compared with muscle pathology findings

Myositis-specific autoantibodies are likely to be associated with distinct cutaneous manifestations ( Table 1). In the case of anti-MDA5 antibody-associated DM, cutaneous ulceration due to vascular injuries was related to rapidly progressive ILD ^32, 33^ and palmar violaceous macules/papules ^32, 34^, in which vasculopathy in the medium and small dermal vessels was frequently observed ^32^.

Severe cutaneous manifestations, including V-neck sign, shawl sign, heliotrope rash, Gottron’s papules/sign, and flagellate erythema, are often observed in patients with anti-TIF1γ antibody-associated DM ^16, 17^. Fiorentino *et al*. termed these characteristic cutaneous manifestations palmar hyperkeratotic papules, psoriasis-like lesions, and hypopigmented and “red on white” telangiectatic patches ^22^.

Mechanic’s hands, characterized by keratotic erythema on the sides of the thumbs and forefingers ^35^, are generally specific to patients with anti-synthetase syndrome, including those with anti-ARS antibody-associated DM ^26^.

Juvenile and adult myopathy patients positive for anti-NXP2 antibody have a high risk of calcinosis ^36^, although patients positive for anti-NXP2 antibody include those with JPM/PM. In contrast, anti-SAE antibody-positive patients with DM demonstrated extensive rash, including erythroderma with “angel wings” sign ^31^.

**Figure 1.**
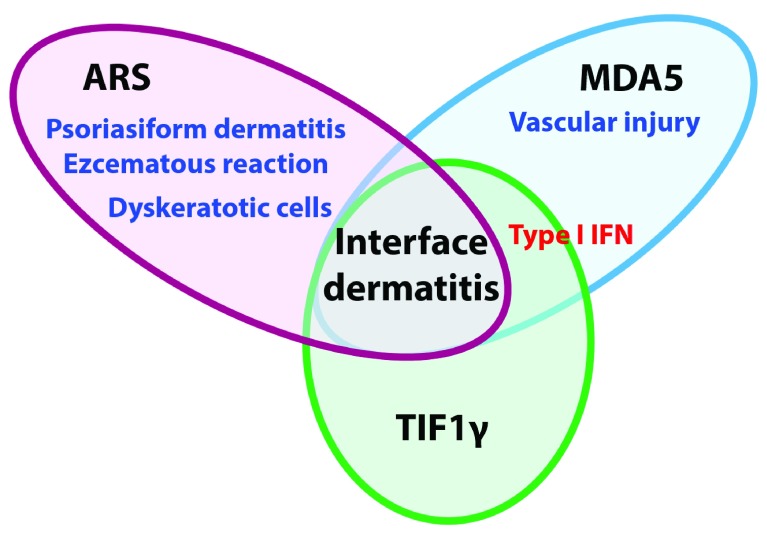
Histopathological classification of skin eruptions in myositis-specific autoantibody-associated groups. The anti-aminoacyl-transfer RNA synthetase (ARS) antibody-positive dermatomyositis (DM) group is characterized by a mixture of psoriasiform dermatitis and eczematous reaction with interface dermatitis mainly presenting dyskeratotic cells and without epidermal expression of myxovirus resistance A (MxA). Vascular injury in the upper dermis and high epidermal expression of MxA are observed in patients with anti-melanoma differentiation-associated protein 5 (anti-MDA5) antibody-positive DM. Epidermal expression of MxA is also detected in patients with anti-transcriptional intermediary factor 1γ (TIF1γ) antibody-positive DM. IFN, interferon.

The histopathological findings of cutaneous lesions in DM include vacuolar degeneration of the basilar keratinocytes, lymphocytic inflammatory infiltrate around the dermal blood vessels, and interstitial mucin deposition. We previously analyzed the histological findings of finger lesions characterized according to myositis-specific autoantibodies (anti-ARS, anti-MDA5, and anti-TIF1γ) ^37^. Our study included finger skin specimens from 30, 19, and 25 cases positive for anti-ARS, anti-MDA5, and anti-TIF1γ antibodies classified according to cutaneous histopathological classifications—(i) interface dermatitis, (ii) psoriasiform dermatitis, (iii) eczematous reaction, and (iv) vascular injury—and also analyzed by immunohistochemistry to detect myxovirus resistance A (MxA) expression, which is usually associated with type I IFN activity. Finger eruptions of anti-ARS antibody-positive DM were histologically characterized by not only interface dermatitis but also psoriasiform dermatitis and eczematous reaction, which were rarely observed in the other patients with DM. Dyskeratotic cells were frequently observed in anti-ARS antibody-positive DM, while vascular injury in the upper dermis was found in anti-MDA5 antibody-positive DM. MxA expression in the epidermis was high in anti-MDA5 antibody-positive DM and rarely observed in anti-ARS antibody-positive DM. The conclusion is shown in Figure 1. MxA expression was rarely observed in the muscle biopsy samples. Previous studies also identified anti-synthetase syndrome as a histological subset in muscle biopsy samples among patients with idiopathic inflammatory myositis, which was characterized by perifascicular necrosis ^38^ and negative MxA expression, which is generally highly expressed in the muscle fibers of patients with DM ^39^. Moreover, a recent study reported that plasma IFN-α levels and the expression of IFN-inducible molecules from peripheral blood mononuclear cells and skin biopsies were higher in anti-MDA5 antibody-associated DM patients than those in anti-ARS antibody-associated or autoantibody-negative DM patients ^40^. Collectively, our findings indicate that these histological characteristics are shared between skin, muscle, and blood samples of patients with DM; that anti-ARS antibody-positive patients are clearly distinguished from other DM subgroups; and that the pathogenesis of anti-MDA5 antibody-associated DM is mediated mainly by type I IFN.

Further studies are needed to clarify the differences among the DM subgroups according to myositis-specific autoantibodies and to provide a basis for the development of subgroup-specific DM therapies.

## Abbreviations

ARS, aminoacyl-transfer RNA synthetase; CADM, clinically amyopathic dermatomyositis; DM, dermatomyositis; IFN, interferon; ILD, interstitial lung disease; JDM, juvenile dermatomyositis; tripartite motif, JPM, juvenile polymyositis; MDA5, melanoma differentiation-associated protein 5; MxA, myxovirus resistance A; PM, polymyositis; RP-ILD, rapidly progressive interstitial lung disease; SAE, small ubiquitin-like modifier activating enzyme; TIF1γ, transcriptional intermediary factor 1γ; TRIM; NXP2, nuclear matrix protein 2
